# Yupingfeng Granule Improves Th2-Biased Immune State in Microenvironment of Hepatocellular Carcinoma through TSLP-DC-OX40L Pathway

**DOI:** 10.1155/2020/1263053

**Published:** 2020-04-09

**Authors:** Fei Yao, Qin Yuan, Xiudao Song, Liang Zhou, Guoqiang Liang, Guorong Jiang, Lurong Zhang

**Affiliations:** Central Laboratory of Suzhou TCM Hospital Affiliated to Nanjing University of Chinese Medicine, Clinical Pharmaceutical Laboratory of Traditional Chinese Medicine, Suzhou Academy of Wumen Chinese Medicine, Suzhou, Jiangsu 215000, China

## Abstract

The tumor immunological microenvironment in hepatocellular carcinoma (HCC) is the T-helper (Th) 2 dominant inhibition state. Improving the immunosuppressive tumor microenvironment represents an important strategy for HCC treatment. TSLP-OX40L pathway is a target to improve Th2 immunosuppression. Yupingfeng granule (YPF) is clinically used to effectively improve the immune status of HCC. In this study, YPF increased the percentage of mature dendritic cells (DCs) and decreased levels of TSLP, TSLPR, and OX40L in tumor and adjacent tissues of the orthotopic-HCC mice model. This occurs together with the decreased levels of Th2 cytokines and increased levels of Th1 cytokines and Th1/Th2 ratio. *In vitro* experiment showed that YPF not only increased the percentage of mature DCs and stimulated IL-12 secretion in DCs but also reduced the positive rate of OX40L expression, decreased the proportion of CD4^+^ IL-13^+^ T cells, increased levels of Th1 cytokines, and decreased levels of Th2 cytokines from TSLP-treated DCs. In summary, these findings demonstrated that YPF promoted the maturation of DCs, decreased OX40L in TSLP-induced DCs, and improved the immunosuppressive state of Th2 in HCC microenvironment. Our results suggest that the mechanism underlying the improving effect of YPF on the immunosuppression is related to the DC-mediated TSLP-OX40L pathway.

## 1. Introduction

Tumor microenvironment in the chronic inflammatory states makes infiltrated immune cells differentiate along the direction of growth, invasion, and metastasis of tumor, thus accelerating the progression of tumor and immune escape processes [1]. The tumor immunological microenvironment in hepatocellular carcinoma (HCC) is the T-helper (Th) 2 dominant inhibition state [2]. Improving the immunosuppressive tumor microenvironment in HCC represents an important strategy for HCC treatment.

Thymic stromal lymphopoietin (TSLP) is a pleiotropic cytokine and activates dendritic cells (DCs) by its receptor (TSLPR). TSLP-activated DCs prime for Th2 cell differentiation. And a variety of studies demonstrated that TSLP could affect Th2 inflammation to participate in the occurrence and development of diseases [3, 4]. OX40, a costimulatory receptor, is transiently expressed on activated T cells [5]. OX40L, the ligand of OX40, is directly regulated by TSLP that is generated by epithelial cells, mast cells, and DCs engaged in Th2 responses [6]. TSLP-activated DCs have the overexpression of costimulatory molecules (i.e., CD80/CD86 and MHC class-II), which leads CD4^+^ T cells to display the phenotypes of Th2 differentiated cells (production of IL-4, IL-5, and IL-13) and upregulates the OX40L expression [7, 8]. OX40L lost the ability to polarize Th2 cells in the presence of IL-12. OX40L functions as the Th2-cell-polarizing signal derived from TSLP-activated DCs [9]. TSLP-OX40L signaling pathway mediated by Th2 responses is involved in human inflammation and immune system [10]. The effects of TSLP on tumor microenvironments of tumor including lung cancer, breast cancer, and pancreatic cancer had been carried out step by step in previous studies. TSLP overexpression was found in tumor tissues of lung cancer and pancreatic cancer. TSLP is almost limited to express in inflammatory sites and activates DCs to promote Th2 immune responses and produces the immunosuppressive factors such as IL-10 and IL-13 [11–13]. Thus, TSLP overexpression and its pathways play an important role in inducing Th2-immunosuppressive state in tumor microenvironment. However, whether TSLP expresses in HCC microenvironment is still unknown. Interestingly, our previous study has found that TSLP, TSLPR, and OX40L are highly expressed in HCC microenvironment [14], which is associated with immunosuppressive status. Therefore, it is likely that the TSLP-OX40L pathway is a target to improve Th2 immunosuppression in HCC microenvironment.

Yupingfeng granule (YPF), an ancient traditional Chinese medicine (TCM) formula, is composed of Astragali Radix (AR; Huangqi; the root of *Astragalus membranaceus* (Fisch.) Bunge), Atractylodis Macrocephalae Rhizoma (AMR; Baizhu; the rhizomes of *Atractylodes macrocephala* Koidz.), and Saposhnikoviae Radix (SR; Fangfeng; the roots of *Saposhnikovia divaricate* (Turcz.) Schischk.). YPF is used for preventing cold and treat upper respiratory tract infection in clinical practice and also used for treatment of immunological diseases such as chronic bronchitis, allergic rhinitis, and asthma [15, 16]. In clinical use for treatment of HCC, the antineoplastic property of YPF could improve the life quality, reduce the malignancy, and enhance the immunity [17–19]. Moreover, a previous study by our group has shown that YPF could improve the immune disorder in mice bearing with HCC by reducing the proportion of CD4^+^ CD25^+^ regulatory T cells, promoting the activation of CD8^+^ T cells, and increasing the proportion of memory CD8^+^ T cells. YPF exerts a therapeutic effect on HCC by improving the immunosuppressive tumor microenvironment. Surprisingly, we found that YPF could significantly reduce the TSLP expression and restore Th1/Th2 balance in tumor and adjacent tissues [16, 20–22]. However, whether YPF regulates the TSLP-OX40L pathway to promote normal maturation of DCs, thereby improving the immunosuppressive tumor microenvironment and exerting an antitumor effect on HCC remains unknown. This study demonstrated that YPF increased the expression levels of DC surface markers, decreased OX40L expression in DCs, and reduced the differentiation of T cells into Th2 cells. Our results suggest that the mechanism underlying the improving effect of YPF on the immunosuppression of tumor microenvironment in HCC is related to the DC-mediated TSLP-OX40L pathway.

## 2. Materials and Methods

### 2.1. Cell Line, Animals, and Reagents

Murine HCC cell line Hepa1-6 was gifted from Dr. Limin Zheng (School of Life Sciences, Sun Yat-sen University). The cells were maintained in Dulbecco's modified Eagle's medium (DMEM) with high glucose (Gibco, Grand Island, NY, USA) supplemented with 10% heat-inactivated fetal bovine serum (Gibco, Grand Island, NY, USA) at 37°C in a humidified atmosphere containing 5% CO_2_.

Male C57/BL6 mice (20–22 g and 6–8 weeks of age) were obtained from the animal experiment center of Matt Albert Technology Co. Ltd. in Suzhou, China (Animal certificate no: SCXK (JING) 2014–0004). The animals were maintained in laminar-flow cabinets under pathogen-free conditions. All the experimental protocols and procedures were in accordance with the EU Directive 2010/63/EU for animal experiments. The study was approved by the Ethics Committee of Suzhou Chinese Medicine Hospital (Suzhou, China).

Yupingfeng granule (5 g/package) was supplied by Guangdong Medi-world Pharmaceutical Co., Ltd. (Guangdong, China). The Chinese medicine granules were manufactured in strict accordance with the standards of Pharmacopoeia of People's Republic of China (2015 edition, Chinese Medical Science and Technology Press).

### 2.2. Animal Model and Treatment

C57/BL6 mice were used for the study after three days of adaptive feeding. The orthotopic transplanted model of murine HCC was established according to our previous study [23]. The HCC murine model was induced by intrahepatic implantation of 1 × 10^6^ Hepa1-6 cells into the left liver lobes of mice, and the animals were maintained in laminar-flow cabinets under pathogen-free conditions. After ten days, the mice were at the initial stage of the tumor and were randomly distributed into five groups (eight mice in each group). Mice in YPF-treated groups were treated with 10 g·kg^−1^, 15 g·kg^−1^, and 20 g·kg^−1^ YPF in 0.2 ml (dissolved in saline) via intragastric administration. Saline served as the model control group, and cisplatin (10 mg/bottle, Qilu Pharmaceutical Co. Ltd., Shandong, China) served as the chemotherapy control once every other day for 2 weeks. Meanwhile, eight C57BL/6 mice without tumor cell inoculation were treated with saline as the normal control group.

Twenty-four hours after the last administration at day 14, the mice were sacrificed. The tumor and adjacent tissues were weighed and then collected. Tumor inhibition rate (%) = (mean tumor weight of the model control group − mean tumor weight of the experiment group)/mean tumor weight of the model control group × 100%.

### 2.3. Preparation of the Homogenate in Tumor and Adjacent Tissues

Tumor and adjacent liver tissue specimens of HCC-bearing mice treated with saline or YPF were homogenized on ice with phosphate buffer (PBS, 50 mg tissue and 500 *μ*l PBS). Homogenates were centrifuged at 3,000 ×*g* for 10 min at 4°C, and the supernatants (100 *μ*l) were used for analysis.

### 2.4. Cytometric Bead Array (CBA) and ELISA Assays of TSLP/TSLPR/OX40L Expression in the Homogenate

To measure cytokine levels, the mouse CBA flex kits were used. IL-2, IL-4, IL-5, IL-10, IL-12p70, IL-13, TNF-α, and IFN-*γ* (BD Biosciences, San Jose, California, USA) cytokines were detected in a single sample. The assays were performed according to the manufacturer's instructions. Samples and standards were acquired on the BD Accuri C6 flow cytometer (BD Biosciences, San Jose, California, USA), and the generated FSC files were analyzed using FCAP Array version 3.0 software (BD Biosciences, San Jose, California, USA).

The concentrations of TSLP/TSLPR/OX40L (R&D Systems, Minneapolis, MN, USA) in the tissue homogenate were quantitatively measured using the commercially available ELISA kits according to manufacturer's protocols.

### 2.5. Preparation of Single-Cell Suspension in Tumor and Adjacent Tissues and Flow Cytometry Detection

The tumor and adjacent tissues were collected and then were cut into small pieces. These pieces were digested at room temperature for 2 h with Hanks solution containing 1 mg·ml^−1^ type-II collagenase. The cells were filtered with the 200 *μ*m pore size microporous membrane and then were washed with PBS twice. DCs were sorted and purified by anti-CD11c microbead isolation kits (Miltenyi Biotec, Auburn, CA, USA) according to the manufacturer's protocol. After cell sorting, DCs were detected by flow cytometry for the markers CD80, CD86, and MHC-II (eBioscience, Grand Island, NY, USA).

### 2.6. Culture and Maturation of Bone Marrow-Derived DCs

The method used for the culture of mouse DCs was reported in a previous study [24]. Bone marrow cells from femur and tibia specimens were flushed out with 10 ml of RPMI-1640 (Gibco, Grand Island, NY, USA) using a syringe and filtrated with a 200 mesh filter. After lysis of red blood cells, the remaining cells were washed with PBS twice. Bone marrow cells were cultured in dishes containing the RPMI-1640 medium with 10% FBS, 20 ng·ml^−1^ recombinant murine GM-CSF (Peprotech, Rocky Hill, NJ, USA), and 20 ng·ml^−1^ recombinant murine IL-4 (Peprotech, Rocky Hill, NJ, USA) at 37 C in a humidified atmosphere containing 5% CO_2_. Every other day, half of the culture medium was replaced with the fresh medium containing GM-CSF and IL-4. On day 6, LPS (Sigma, St. Louis, MO, USA), YPF, recombinant mouse TSLP (R&D Systems, Minneapolis, MN, USA), and TSLP with YFP were added into the culture. The semiadherent DCs were harvested on day 7 and detected by flow cytometry for the markers CD80, CD86, MHC-II, and CD252 (OX40L) (eBioscience, Grand Island, NY, USA). Cytokine (IL-12p70) concentrations in cell culture supernatants were measured with ELISA kits (R&D Systems, Minneapolis, MN, USA).

### 2.7. Mix Lymphocyte Reaction

This assay evaluated the stimulating effect of DCs on T cells. DCs cocultured with TSLP 24 h were used as stimulator cells. T lymphocytes were obtained from C57/BL6 mice using the lymphocyte separation medium (Ficoll-Paque PLUS, GE, MA, USA). The stimulated DCs and T cells were cocultured in 6-well flat-bottom plates at a ratio of 1 : 10 in the RPMI-1640 medium with 10% FBS as described previously [25]. The different concentrations of YPF were added in the mixed lymphocyte for 48 h. The collected cells were detected by flow cytometry for CD4 and IL-13 (eBioscience, Grand Island, NY, USA). The collected culture supernatants were used for cytokine level assessment with CBA flex kits (BD Biosciences, San Jose, California, USA).

### 2.8. Statistical Analysis

All data represent at least three independent experiments, and results of the experimental studies are expressed as mean ± standard deviation (SD). Statistical significance of differences was analyzed by the Student's *t*-test or one-way analysis of variance followed by the Bonferroni or Dunnett's post hoc tests (Graph Pad Prism Software, San Diego, CA, USA). The values of *p* < 0.05 were considered statistically significant.

## 3. Results

### 3.1. YPF Inhibited the Tumor Growth and Improved Th2-Biased Immune State of Tumor Microenvironment in Mice Bearing with HCC

**Figure 1:**
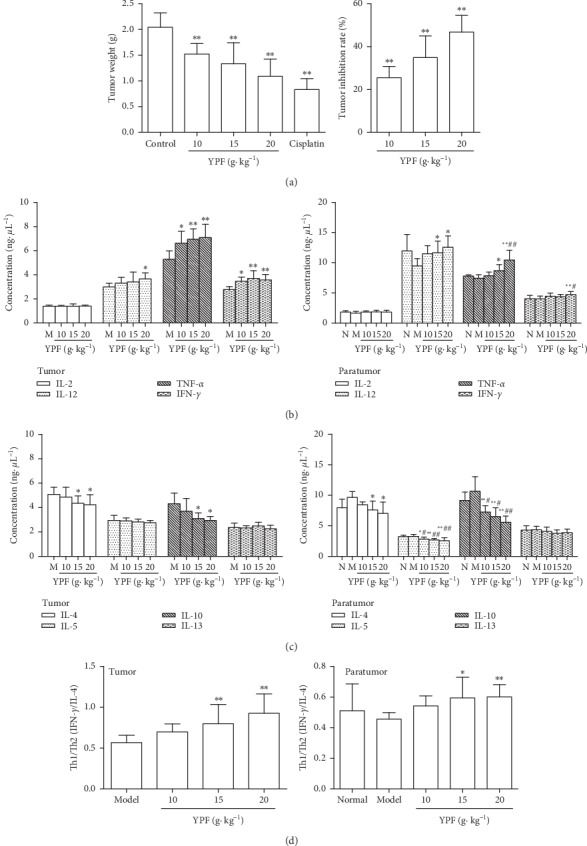
YPF inhibited the tumor growth and improved Th2-biased immune state of the tumor microenvironment in mice bearing with HCC. (a) Tumor weights of the C57/BL6 mice inoculated with Hepa1-6 cells and treated with YPF 14 days to calculate the tumor inhibition rate of YPF. (b, c, and d) Effect of YPF on Th1 cytokine levels, Th2 cytokine levels, and Th1/Th2 ratio in tumor and adjacent tissues of tumor-bearing mice. ^*∗*^*p* < 0.05 , ^*∗∗*^*p* < 0.01 vs. model control group; ^#^*p* < 0.05 , ^##^*p* < 0.01 vs. normal control group.

After the last administration (food deprivation for 12 h), the mice were weighed and sacrificed. Tumor and adjacent tissues were isolated from tumor-bearing mice, and homogenate was prepared. Cytokine contents were detected by flow cytometry with the CBA kit. Compared with the model control group, YPF increased the expression of IL-12, TNF-*α*, and IFN-*γ* in tumor and adjacent tissues (*p* < 0.05 and *p* < 0.01) but did not affect IL-2 level (*p* > 0.05). Compared with the normal control group, the levels of Th1 cytokines (IL-2, IL-12, TNF-*α*, and IFN-*γ*) in the adjacent tissues of the model control group were on a downward trend, but there was no statistical significance (*p* > 0.05). YPF (20 g·kg^−1^) increased the expression of TNF-*α* and IFN-*γ* in adjacent tissues (*p* < 0.05, *p* < 0.01; Figure 1(b)). It was suggested that YPF could improve the overall level of Th1 cytokines in tumor and adjacent tissues of tumor-bearing mice.

Compared with the model control group, YPF decreased the expression of IL-4 and IL-10 in tumor tissues and adjacent tissues (*p* < 0.05 and *p* < 0.01) and IL-5 in adjacent tissues (*p* < 0.05 and *p* < 0.01) but had no effect on IL-13 (*p* > 0.05). Compared with the normal control group, the levels of Th2 cytokines (IL-4, IL-5, IL-10, and IL-13) in the adjacent tissues of the model control group showed no significant change (*p* > 0.05). But, YPF significantly decreased the expression of IL-5 and IL-10 in adjacent tissues (*p* < 0.05, *p* < 0.01; Figure 1(c)). It was suggested that YPF could reduce the overall level of Th2 cytokines in tumor and adjacent tissues of tumor-bearing mice.

Based on the study of the changes of Th1 and Th2 cytokines, the Th1/Th2 ratio was used to reflect the immune status of tumors and adjacent tissues. IFN-*γ*/IL-4 value often represents the Th1/Th2 balance. Compared with the model control group, the ratio of Th1/Th2 (IFN-*γ*/IL-4) in tumors and adjacent tissues was significantly increased in YPF groups (15 and 20 g kg^−1^) (*p* < 0.05, *p* < 0.01; Figure 1(d)). It was suggested that YPF could adjust the Th2 dominant status in tumors and adjacent tissues of tumor-bearing mice.

The results also showed that YPF could inhibit the growth of HCC in a dose-dependent manner. Compared with the model control group, YPF (10, 15, and 20 g·kg^−1^) treatment significantly suppressed tumor growth (*p* < 0.01). Tumor inhibitory rates for YPF (10, 15, and 20 g kg^−1^) were 25.48%, 34.92%, and 46.76% (Figure 1(a)). These results mainly indicated that YPF may inhibit the growth of tumors by adjusting Th2-biased in the state of tumors.

### 3.2. YPF Affected the TSLP-OX40L Pathway and DC Maturation on Tumor Microenvironment in Mice Bearing with HCC

**Figure 2:**
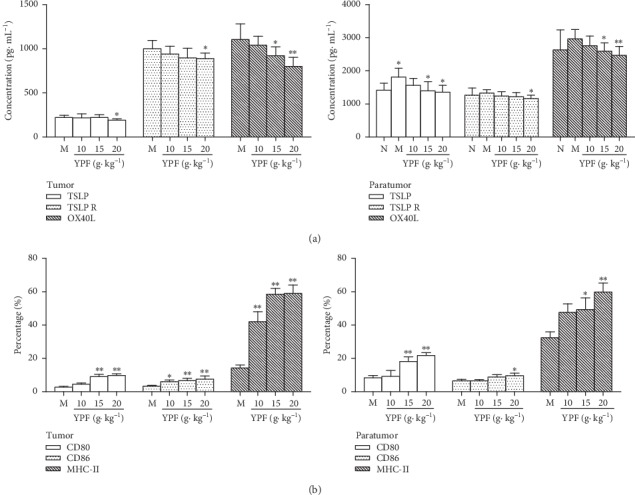
YPF affected the TSLP-OX40L pathway and DC maturation on tumor microenvironment in mice bearing with HCC. (a) Effect of YPF on TSLP, TSLPR, and OX40L levels in tumor and adjacent tissues of tumor-bearing mice. (b) Effect of YPF on CD80, CD86, and MHC-II of DCs in tumor and adjacent tissues of tumor-bearing mice. ^*∗*^*p* < 0.05, ^*∗∗*^*p* < 0.01 vs. model control group.

TSLP is a key factor mediating the Th2 reaction at the interface between the body and the environment (skin, intestine, respiratory tract, etc.). Activating DCs through TSLPR can upregulate the expression of the OX40 ligand (OX40L) in DCs [26, 27], thereby inducing Th2 inflammatory response [28]. Therefore, we further observed the expression of TSLP, TSLPR, and OX40L in tumor and adjacent tissue after administration of YPF. The results showed that the levels of TSLP, TSLPR, and OX40L in the adjacent tissues of the model control group were higher than those of the normal control group. Among these variations, a statistically significant difference in TSLP was found (*p* < 0.05). Compared with the model control group, YPF decreased the levels of TSLP, TSLPR, and OX40L in tumor and adjacent tissues (*p* < 0.05, *p* < 0.01; Figure 2(a)). It was suggested that YPF could decrease the levels of TSLP, TSLPR, and OX40L of tumor microenvironment in mice bearing with HCC.

At the same time, we also observed the maturation of DC cells in tumor and adjacent tissues. Single-cell suspensions of DCs were stained for the markers CD80, CD86, and MHC-II. Compared with the model control group, YPF increased the expression of CD80, CD86, and MHC-II of DCs in tumors and adjacent tissues (*p* < 0.05, *p* < 0.01; Figure 2(b)). It showed that YPF could increase the percentage of mature DCs and promote the maturation of DCs in tumor microenvironment in mice bearing with HCC, thereby adjusting the immunosuppressive state of the Th2 type.

### 3.3. YPF Effect on Maturation and IL-12 Secretion of DCs *In Vitro*

**Figure 3:**
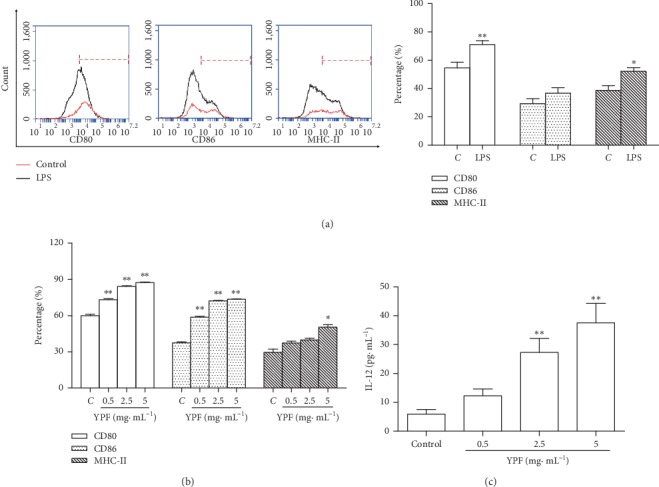
YPF effect on maturation and IL-12 secretion of DCs *in vitro*. (a) Immature DCs derived from the bone marrow of mice were cultured and differentiated for 6 days. LPS was added to stimulate the maturation of immature DCs. (b) Effect of YPF on CD80, CD86, and MHC-II on the DC surface. (c) Effect of YPF on IL-12 secreted by DCs. ^*∗*^*p* < 0.05, ^*∗∗*^*p* < 0.01 vs. control group.

Further *in vitro* experiments were carried out. The effects of YPF on the differentiation and maturation of DCs derived from the bone marrow of mice (CD80, CD86, and MCH-II expression) and the secretion level of IL-12 were observed. The results showed that immature DCs were cultured and differentiated for 6 days. LPS (1 *μ*g·mL^−1^) was added for 24 h to stimulate the maturation of immature DCs. Compared with the control group, LPS increased the expression levels of CD80, CD86, and MHC-II (*p* < 0.05, *p* < 0.01; Figure 3(a)). YPF increased the expression levels of CD80, CD86, and MHC-II (*p* < 0.05, *p* < 0.01; Figure 3(b)) and stimulated IL-12 secretion (*p* < 0.01; Figure 3(c)) in DCs. It was further confirmed that YPF could stimulate the maturation of DCs *in vitro*.

### 3.4. YPF Effect on TSLP-Mediated DCs *In Vitro*

**Figure 4:**
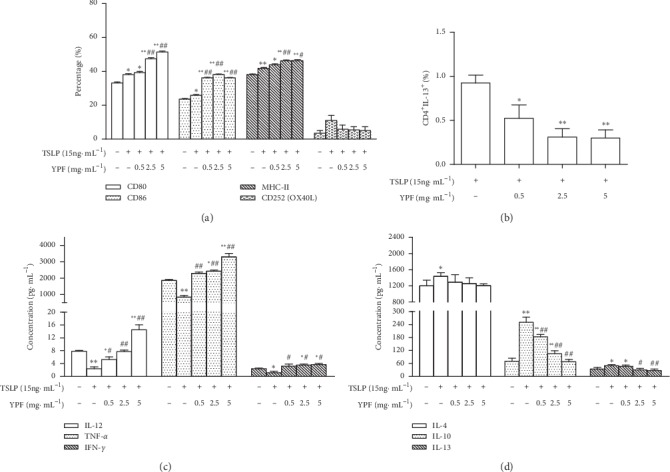
YPF effect on TSLP-mediated DCs *in vitro*. (a) Effect of YPF on CD80, CD86, MHC-II, and CD252 (OX40L) on the TSLP-mediated DC surface. (b) Effect of YPF on differentiation of CD4^+^ IL-13^+^ T cells in the mixed lymphocyte reaction. (c and d) Effect of YPF on Th1 and Th2 cytokine levels in culture supernatants of the mixed lymphocyte reaction. ^*∗*^*p* < 0.05, ^*∗∗*^*p* < 0.01 vs. control group; ^#^*p* < 0.05, ^##^*p* < 0.01 vs. TSLP group.

As TSLP is a potent activator of DCs [25], we evaluate whether YPF improves the mature states of DCs induced by TSLP. First, cell-surface receptors of DCs are required for T-cell activation [29]. Compared with the control group, both TSLP and YPF increased the expression levels of CD80, CD86, and MHC-II (*p* < 0.05, *p* < 0.01; Figure 4(a)). As DCs express OX40L, a Th2-polarizing molecule [30], we further examined the ability of TSLP to activate DCs to produce OX40L. Overexpression of CD252 (OX40L) was found in TSLP-induced DCs, and YPF decreased the positive expression rate (Figure 4(a)).

Additionally, TSLP induces the expression of maturation markers on DCs and increases their ability to polarize lymphocytes into the Th2 phenotype [31]; we assessed the capability of DCs in different groups to stimulate and polarize T-cell response by coculturing them with naïve T cells, an assay used to evaluate the stimulatory ability of DCs to alter T-cell differentiation and cytokine production. Compared with the mix control group, YPF reduced the proportion of CD4^+^ IL-13^+^ T cells (*p* < 0.05, *p* < 0.01; Figure 4(b)). Compared with the mix control group, TSLP decreased the expression of Th1-type cytokines (IL-12, TNF-*α*, and IFN-*γ*, *p* < 0.05, *p* < 0.01) and increased the expression of Th2-type cytokines (IL-4, IL-10, and IL-13, *p* < 0.05, *p* < 0.01). DC-T-cell supernatants in the YPF groups showed significantly high levels of IL-12, TNF-*α*, and IFN-*γ* (*p* < 0.05, *p* < 0.01) and lower levels of IL-10 and IL-13 (*p* < 0.05, *p* < 0.01), while IL-4 shows a downward trend but has no statistical significance compared with the TSLP group (Figures 4(c) and 4(d)). These findings demonstrated that YPF promoted the expression levels of TSLP-induced DC surface markers, decreased OX40L in TSLP-induced DCs, changed the differentiation of T cells into Th2 cells, and affected the potential of DCs mediated by TSLP to polarize lymphocytes.

## 4. Discussion

In this study, in the orthotopic-mice model of HCC, YPF increased the levels of Th1 cytokines (IL-2, IL-12, TNF-*α*, and IFN-*γ*), decreased the levels of Th2 cytokines (IL-4, IL-5, IL-10, and IL-13), and increased the Th1/Th2 ratio in tumor and adjacent tissues. It suggests that YPF exerts anti-HCC effect by improving the Th2-immunosuppressive state. Our data also showed that YPF decreased the levels of TSLP, TSLPR, and OX40L, increased the expression of CD80, CD86, and MHC-II in DCs, and stimulated the maturation of DCs in tumor and adjacent tissues of mice bearing with HCC. Meanwhile, YPF increased the expression of CD80, CD86, and MHC-II and the secretion of IL-12 in DCs *in vitro*. YPF also reduced the positive expression rate of CD252 (OX40L), decreased the proportion of CD4^+^ IL-13^+^ T cells, increased the levels of Th1 cytokines (IL-12, TNF-*α*, and IFN-*γ*), and decreased the levels of Th2 cytokines (IL-4, IL-10, and IL-13) from TSLP-induced DCs. These findings demonstrated that YPF promotes the expression levels of DC surface markers, decreases OX40L in DCs, changes the differentiation of T cells into Th2 cells, and affects the potential of DCs mediated by TSLP to polarize lymphocytes. Our results suggest that the mechanism underlying the improving effect of YPF on the immunosuppression of tumor microenvironment in HCC is related to the DC-mediated TSLP-OX40L pathway.

Th1 and Th2 lymphocytes are the two main subsets of CD4 T-helper cells with different functions and patterns of cytokine secretion, which are identified in both mice and humans. Th1 and Th2 are in a state of dynamic balance, maintaining the normal immune function of the body. Th1/Th2 bias is different with different immune statuses. Th1 cells mainly secret cytokines such as IL-2, IL-12, TNF-*α*, and IFN-*γ*, which can enhance the cytotoxicity of immune cells to kill tumor cells and make the body's anticancer immune response stronger [32, 33]. Th2 cells mainly secrete cytokines such as IL-4, IL-5, IL-10, and IL-13, which are involved in humoral immunity. It is generally believed that Th2 cytokines can inhibit the differentiation of CD4^+^ T cells into Th1 cells and weaken the immune response to antitumor effect, thus promoting the development of tumors [34, 35]. Downregulated Th1 and upregulated Th2 cytokine production has been found in tumor and adjacent tissues of HCC patients. It suggests that Th1/Th2 shift exists in the immunosuppressive state [33]. In the present study, the levels of Th1 cytokines (IL-2, IL-12, TNF-*α*, and IFN-*γ*) were on a downward trend, but the levels of Th2 cytokines (IL-4, IL-5, IL-10, and IL-13) were increased. The ratio of Th1/Th2 (IFN-*γ*/IL-4) was on a downward trend in the adjacent tissues of mice bearing with HCC. These results showed that the tumor microenvironment of HCC was Th2-dominanted immunosuppression. YPF, a classical TCM formulation, has been traditionally used for treatment of immune system-related diseases such as chronic bronchitis, allergic rhinitis, and asthma [36]. A previous study has demonstrated that YPF efficiently inhibited Th2 cytokines [15]. The current study showed that YPF could improve the immune suppression caused by the imbalance of Th1/Th2 ratio in the tumor microenvironment of HCC. It suggests that YPF exerts anti-HCC effect by improving the Th2 immunosuppressive state.

TSLP is a member of the 4-helix bundle cytokine family, and a distant paralog of IL-7 is first identified as an activity in the supernatants of a mouse thymic stromal cell line. It has now become apparent that a major TSLP-responsive cellular subset in both humans and mice is myeloid-derived dendritic cells (mDCs) [37]. Pedroza-Gonzalez showed that inflammatory Th2 cells that promoted tumor development were driven by cancer cell-derived TSLP, which induced and maintained OX40L-expressing DCs in the tumor microenvironment. TSLP induces DC maturation through combination with TSLPR on the surface of DCs. The maturing DCs can upregulate the expression of OX40L through TSLPR. And mature DCs then promote Th2 differentiation of naïve CD4 T cells and induce CD4^+^ T cells to secrete type 2 cytokines such as IL-13, contributing to tumor development [28, 38]. In our experiment *in vitro*, a similar phenomenon was observed. TSLP promoted the maturation of DCs, increased the expression of OX40L, differentiated CD4^+^ IL-13^+^ T cells, decreased the secretion of Th1 cytokines, and increased the secretion of Th2 cytokines. Interestingly, YPF can promote the maturation of DCs but reduce the expression of OX40L (CD252) induced by TSLP. However, YPF significantly reduced the proportion of CD4^+^ IL-13^+^ T cells, increased the secretion of Th1 cytokines (IL-12, TNF-*α*, and IFN-*γ*), and decreased the secretion of Th2 cytokines (IL-4, IL-10, and IL-13) in DCs induced by TSLP. Our data also showed that YPF decreased the levels of TSLPR and OX40L in tumor and adjacent tissues *in vivo*. We speculated that the decrease of the OX40L expression was related to the decrease of the TSLPR expression. It was further confirmed that the mechanism of YPF in improving tumor microenvironment in mice with HCC was related to the TSLP-OX40L pathway.

Recently, a new and unexpected function for TSLP has been found for both promotion and suppression of solid tumor growth [39]. In mouse models of breast and pancreatic carcinogenesis, it was found that early administration of TSLP blocked cancer development [40]. But, in the middle and the late stage, the level of the TSLP expression was correlated with tumor growth and metastasis [12], and tumor-derived TSLP could act on TSLPR^+^ endothelial cells to promote angiogenesis in cervical cancer [41]. Conversely, Di Piazza and Demehri demonstrated that TSLP exerted a tumor-suppressing role in the murine model of skin cancer [26, 40]. Yue et al. also reported the decreased TSLP expression in human colon cancer and TSLP levels was negatively correlated with the clinical staging score of cancer, and TSLP enhanced apoptosis of colon cancer cells through the engagement of TSLPR [42]. The present study shows for the first time that the expressions of TSLP, TSLPR, and OX40L, which were the key target proteins of the TSLP-OX40L pathway, were increased in the tumor microenvironment of mice bearing with HCC. The TSLP expression in HCC was positively correlated with its microenvironment immunosuppression. But, the different roles of TSLP in early and advanced HCC are deserved for further study.

## 5. Conclusions

We present the first evidence of TSLP protein expression in tumor and adjacent tissues of HCC. Our results suggest that the TSLP-OX40L pathway is involved in the immunosuppression of HCC microenvironment, and YPF is an immunomodulator against the microenvironment immune suppression of HCC in mice. The mechanism underlying the improving effect of YPF in improving the immunosuppression of tumor microenvironment in HCC may be related to the TSLP-OX40L pathway, which provides the experimental and theoretical basis for YPF to prevent and treat HCC. Development of the targeted TSLP pathway to improve the microenvironment immunosuppression of HCC provides a new strategy for prolonging the survival of patients with HCC and new targets for ideal therapeutic drugs.

## Data Availability

The data and materials supporting the conclusions of this article are included within the article.

## References

[1] Wang D., DuBois R. N. (2015). Immunosuppression associated with chronic inflammation in the tumor microenvironment. *Carcinogenesis*.

[2] Chew V., Tow C., Teo M. (2010). Inflammatory tumour microenvironment is associated with superior survival in hepatocellular carcinoma patients. *Journal of Hepatology*.

[3] Hanabuchi S., Watanabe N., Liu Y.-J. (2012). TSLP and immune homeostasis. *Allergology International*.

[4] Ziegler S. F., Roan F., Bell B. D., Stoklasek T. A., Kitajima M., Han H. (2013). The biology of thymic stromal lymphopoietin (TSLP). *Advances in Pharmacology*.

[5] Gauvreau G. M., Boulet L.-P., Cockcroft D. W. (2014). OX40L blockade and allergen-induced airway responses in subjects with mild asthma. *Clinical & Experimental Allergy*.

[6] Kaur D., Brightling C. (2012). OX40/OX40 ligand interactions in T-cell regulation and asthma. *Chest*.

[7] Soumelis V., Reche P. A., Kanzler H. (2002). Human epithelial cells trigger dendritic cell-mediated allergic inflammation by producing TSLP. *Nature Immunology*.

[8] Zhou B., Comeau M. R., Smedt T. D. (2005). Thymic stromal lymphopoietin as a key initiator of allergic airway inflammation in mice. *Nature Immunology*.

[9] Ito T., Wang Y.-H., Duramad O. (2005). TSLP-activated dendritic cells induce an inflammatory T helper type 2 cell response through OX40 ligand. *Journal of Experimental Medicine*.

[10] Meng Q., Liu X., Li P. (2016). The influence of house dust mite sublingual immunotherapy on the TSLP-OX40L signaling pathway in patients with allergic rhinitis. *International Forum of Allergy & Rhinology*.

[11] Li H., Zhao H., Yu J. (2011). Increased prevalence of regulatory T cells in the lung cancer microenvironment: a role of thymic stromal lymphopoietin. *Cancer Immunology, Immunotherapy*.

[12] Olkhanud P. B., Rochman Y., Bodogai M. (2011). Thymic stromal lymphopoietin is a key mediator of breast cancer progression. *Journal of Immunology (Baltimore, Md.: 1950)*.

[13] De Monte L., Reni M., Tassi E. (2011). Intratumor T helper type 2 cell infiltrate correlates with cancer-associated fibroblast thymic stromal lymphopoietin production and reduced survival in pancreatic cancer. *The Journal of Experimental Medicine*.

[14] Yuan Q., Yao F., Zhang L. R. (2016). Research on inhibition effects of Yupingfeng powder through TSLP change the balance of Th1/Th2 in hepatocellular carcinoma. *Journal of Nantong University (Medical Sciences)*.

[15] Shen D., Xie X., Zhu Z. (2014). Screening active components from Yu-ping-feng-san for regulating initiative key factors in allergic sensitization. *PLoS One*.

[16] Du C. Y., Choi R. C., Zheng K. Y. (2013). Yu ping feng san, an ancient Chinese herbal decoction containing Astragali Radix, Atractylodis Macrocephalae rhizoma and Saposhnikoviae Radix, regulates the release of cytokines in murine macrophages. *PLoS One*.

[17] Zhang S. X., Liu H. L., Fan Z., Jin Z. Z., Wang J. J. (2017). Influence of Yupingfeng powder adjuvant therapy on treatment effect and anti-tumor immunity of patients with primary liver cancer. *Anti-tumor Pharmacy*.

[18] Xie C. H., Zeng X. J., Chen H. L. (2017). Effect of Yupingfeng powder on immune function and quality of life of lung cancer patients treated with chemotherapy. *Heilongjiang Medicine and Pharmacy*.

[19] Zhou Y. (2017). Research progress of Yupingfeng powder in tumor prevention and treatment. *World Clinical Drugs*.

[20] Zhang L. R., Yao F., Jiang G. R. (2014). Research on the immunoregulation effects of Yupingfeng decotion on hepatoma-bearing mice. *Journal of Southeast University (Medical Science Edition)*.

[21] Zhang L. R., Yao F., Jiang G. R., Liang G. Q. (2014). Research on direct inhibition and immunoregulation effects of Yupingfeng powder in hepatic cell carcinoma. *Chinese Archives of Traditional Chinese Medcine*.

[22] Yao F., Zhang L. R., Jiang G. R. (2014). Influence of Yupingfeng powder on T lymphocytes phenotype in Hepa1-6 HCC tumor-bearing mice. *Hebei Journal of Traditional Chinese Medicine*.

[23] Yao F., Zhang L. R., Jiang G. R. (2018). Osthole attenuates angiogenesis in an orthotopic mouse model of hepatocellular carcinoma via the downregulation of nuclear factor-*κ*B and vascular endothelial growth factor. *Oncology Letters*.

[24] Hong X., Dong T. G., Yi T. (2018). Impact of 5-Fu/oxaliplatin on mouse dendritic cells and synergetic effect with a colon cancer vaccine. *Chinese Journal of Cancer Research*.

[25] Li H.-T., Chen Z.-G., Lin Y.-S. (2018). CpG-ODNs and budesonide act synergistically to improve allergic responses in combined allergic rhinitis and asthma syndrome induced by chronic exposure to ovalbumin by modulating the TSLP-DC-OX40L axis. *Inflammation*.

[26] Di Piazza M., Nowell C. S., Koch U., Durham A. D., Radtke F. (2012). Loss of cutaneous TSLP-dependent immune responses skews the balance of inflammation from tumor protective to tumor promoting. *Cancer Cell*.

[27] Pattarini L., Trichot C., Bogiatzi S. (2017). TSLP-activated dendritic cells induce human T follicular helper cell differentiation through OX40-ligand. *Journal of Experimental Medicine*.

[28] Sun L., Chen C., Wu J., Dai C., Wu X. (2018). TSLP-activated dendritic cells induce T helper type 2 inflammation in Aspergillus fumigatus keratitis. *Experimental Eye Research*.

[29] Wang Y., Liang Y., Zhang Y. M., Wu D. P., Liu H. Y. (2015). Bortezomib inhibits bone marrow-derived dendritic cells. *International Journal of Clinical & Experimental Pathology*.

[30] Wang W. L., Li H. Y., Zhang M. S. (2013). Thymic stromal lymphopoietin: a promising therapeutic target for allergic diseases. *International Archives of Allergy and Immunology*.

[31] Kopecka J., Rozkova D., Sediva A. (2013). Plasmacytoid DCs, exposed to TSLP in synergy with TLR ligands, acquire significant potential towards Th2 polarization. *Medical Science Monitor Basic Research*.

[32] Cosmi L., Maggi L., Santarlasci V., Liotta F., Annunziato F. (2014). T helper cells plasticity in inflammation. *Cytometry Part A*.

[33] Saxena R., Kaur J. (2015). Th1/Th2 cytokines and their genotypes as predictors of hepatitis B virus related hepatocellular carcinoma. *World Journal of Hepatology*.

[34] Ma C., Kesarwala A. H., Eggert T. (2016). NAFLD causes selective CD4^+^ T lymphocyte loss and promotes hepatocarcinogenesis. *Nature*.

[35] Mitchell R. E., Hassan M., Burton B. R. (2017). IL-4 enhances IL-10 production in Th1 cells: implications for Th1 and Th2 regulation. *Scientific Reports*.

[36] Nikles S., Monschein M., Zou H. (2017). Metabolic profiling of the traditional Chinese medicine formulation Yu Ping Feng San for the identification of constituents relevant for effects on expression of TNF-*α*, IFN-*γ*, IL-1*β* and IL-4 in U937 cells. *Journal of Pharmaceutical and Biomedical Analysis*.

[37] Lo Kuan E., Ziegler S. F. (2014). Thymic stromal lymphopoietin and cancer. *Journal of Immunology (Baltimore, Md.: 1950)*.

[38] Pedroza-Gonzalez A., Xu K., Wu T.-C. (2011). Thymic stromal lymphopoietin fosters human breast tumor growth by promoting type 2 inflammation. *The Journal of Experimental Medicine*.

[39] Varricchi G., Pecoraro A., Marone G. (2018). Thymic stromal lymphopoietin isoforms, inflammatory disorders, and cancer. *Frontiers in Immunology*.

[40] Demehri S., Cunningham T. J., Manivasagam S. (2016). Thymic stromal lymphopoietin blocks early stages of breast carcinogenesis. *Journal of Clinical Investigation*.

[41] Xie F., Meng Y. H., Liu L. B. (2013). Cervical carcinoma cells stimulate the angiogenesis through TSLP promoting growth and activation of vascular endothelial cells. *American Journal of Reproductive Immunology (New York, N.Y.: 1989)*.

[42] Yue W. J., Lin Y. L., Yang X. G. (2016). Thymic stromal lymphopoietin (TSLP) inhibits human colon tumor growth by promoting apoptosis of tumor cells. *Oncotarget*.

